# Source Regions of the First Immigration of Fall Armyworm, *Spodoptera frugiperda* (Lepidoptera: Noctuidae) Invading Australia

**DOI:** 10.3390/insects12121104

**Published:** 2021-12-10

**Authors:** Guo-Jun Qi, Jian Ma, Jing Wan, Yong-Lin Ren, Simon McKirdy, Gao Hu, Zhen-Fei Zhang

**Affiliations:** 1 Guangdong Provincial Key Laboratory of High Technology for Plant Protection, Plant Protection Research Institute, Guangdong Academy of Agricultural Science, Guangzhou 510640, China; qigj@gdppri.com; 2 College of Plant Protection, Nanjing Agricultural University, Nanjing 210095, China; 2017202026@njau.edu.cn; 3 Harry Butler Institute, Murdoch University, Perth 6150, Australia; 33386687@student.murdoch.edu.au (J.W.); Y.Ren@murdoch.edu.au (Y.-L.R.); s.mckirdy@murdoch.edu.au (S.M.)

**Keywords:** *Spodoptera frugiperda*, migration, Australia, trajectory analysis, wind systems

## Abstract

**Simple Summary:**

The fall armyworm, *Spodoptera frugiperda*, is a serious invasive, crop-destroying pest, which is the subject of a global warning from the United Nations Food and Agriculture Organization (FAO). The surprisingly rapid spread of fall armyworm and its significant capacity to generate high-yield losses has attracted increased attention worldwide. In January 2020, fall armyworm was first confirmed in Torres Strait (Saibai and Erub Islands) and mainland Australia (Bamaga). However, the possible source region of the first immigration of fall armyworm was still obscure. A better understanding of the migration paths and the source regions of fall armyworm immigrants, will be useful for the monitoring and early warning of this pest in Australia. The migratory paths and wind systems for the first case of the immigration of fall armyworm invading Australia were simulated using a three-dimensional trajectory analysis program. This study has confirmed that the immigration of fall armyworm to Australia was most likely from Sulawesi and Moluccas Islands of Indonesia.

**Abstract:**

Fall armyworm is recognized as one of most highly destructive global agricultural pests. In January 2020, it had first invaded Australia, posing a significant risk to its biosecurity, food security, and agricultural productivity. In this study, the migration paths and wind systems for the case of fall armyworm invading Australia were analyzed using a three-dimensional trajectory simulation approach, combined with its flight behavior and NCEP meteorological reanalysis data. The analysis showed that fall armyworm in Torres Strait most likely came from surrounding islands of central Indonesia on two occasions via wind migration. Specifically, fall armyworm moths detected on Saibai and Erub Islands might have arrived from southern Sulawesi Island, Indonesia, between January 15 and 16. The fall armyworm in Bamaga most likely arrived from the islands around Arafura Sea and Sulawesi Island of Indonesia, between January 26 and 27. The high risk period for the invasion of fall armyworm is only likely to have occurred in January–February due to monsoon winds, which were conducive to flight across the Timor Sea towards Australia. This case study is the first to confirm the immigration paths and timing of fall armyworm from Indonesia to Australia via its surrounding islands.

## 1. Introduction

The fall armyworm, *Spodoptera frugiperda* (J.E. Smith) (Lepidoptera: Noctuidae), is a highly destructive agricultural pest that is noted for its wide host range, strong long-distance flight capability, and potential to inflict high yield losses [1,2]. This noctuidae is a polyphagous pest with the caterpillar being the damaging stage of the pest [3]. The fall armyworm prefers maize, but can feed on over 350 additional species of host plants [4], including rice, sorghum, sugarcane, wheat, cotton and many vegetable and horticultural crops [5]. The fall armyworm was the subject of a global warning from the United Nations Food and Agriculture Organization (FAO) in August 2018 [6]. It has caused huge economic losses in crop production and posed a real threat to global food security [7].

Due to the increasing global trade and the moth’s long-distance migration ability, the fall armyworm has spread with remarkable speed and caused high yield losses to maize production globally [8,9,10]. Originally, native to the Americas, fall armyworm has long been a major agricultural problem in the Western Hemisphere [1,3]. Since 2016, it has rapidly spread to and throughout the vast region of Africa [11,12], the Indian subcontinent [13], Southeast Asia, and East Asia [14,15]. It is well known that eradicating the fall armyworm can be impossible once it has infested a new region. The fall armyworm, however, is still spreading and expanding its hazardous zone with its long-distance migration [7]. The surprisingly rapid spread of fall armyworm and its significant capacity to generate high yield losses have attracted increased attention worldwide.

Invasion of the fall armyworm has become an unstoppable trend sweeping across the whole world. Fall armyworm infestation has breached Indonesia, Timor-Leste, and Papua New Guinea since 2019 [16]. In January 2020, fall armyworm was confirmed in surveillance traps in the islands of Saibai and Erub in Torres Strait, and then also detected at Bamaga, in northern part of Queensland, which was its first introgression into mainland Australia [17]. Moreover, Australia has sufficient cultivation of suitable host plants, tropical and sub-tropical climate characteristics that could permit fall armyworm invasion and establishment [7,18]. Recent modelling has shown that the patterns of dispersal, which are driven by seasonal winds, are a plausible means of introduction of Culicoides into northern Australia [18]. However, possible source region of the first immigration of adult fall armyworm was still obscure. A better understanding of the possible migration paths and the source regions of the first invasion of fall armyworm, will be useful for the monitoring and early warning of this pest in Australia.

In this study, the evidence of surveillance traps for the first invasion of fall armyworm was reported by morphological and molecular diagnostics. The possible date of the first invasion of fall armyworm was analyzed. The migratory paths and possible source regions for the case of fall armyworm invading Saibai and Erub Islands and Bamaga, Australia were simulated using three-dimensional trajectory analysis program and Weather Research and Forecasting model [19], combined with its self-powered flight behavior of fall armyworm and NCEP meteorological reanalysis data. In addition to the influence on airspeed and orientation on migratory displacement of fall armyworm, unfavorable atmospheric factors or phenomena terminating insect flight, were also considered.

## 2. Materials and Methods

### 2.1. Surveillance Trapping and Possible Immigrating Date

In order to detect the fall armyworm invasion as early as possible, eight surveillance traps using sex pheromone for specific fall armyworm were installed and monitored by the Australian Department of Agriculture, Water and Environment’s Northern Australia Quarantine Strategy (NAQS) since early October, 2019 (Table 1). The moths detected in the surveillance traps must be immigrants due to no trace of fall armyworm in Australia. The specimens were confirmed by morphological and molecular diagnostics. The possible immigrating dates of the first detected fall armyworm can be inferred based on the date of capturing moth with the surveillance trap. However, as the captured fall armyworm moths are not collected daily, the exact trapping dates of the first invasion of fall armyworm should be inferred by favorable atmospheric factors.

### 2.2. Meteorological Data and Modeling

The Weather Research and Forecasting (WRF) Model (version 3.8, https://www2.mmm.ucar.edu/wrf/users/download, accessed on 12 February 2021) is a next-generation mesoscale numerical weather prediction system, which provided the hourly meteorological condition data necessary for trajectory calculation [20,21]. The National Centers for Environmental Prediction (NCEP) Final Analysis (FNL) data produced from the Global Data Assimilation (GDAS) were used as the meteorological data for the model inputs. GDAS FNL is a 6-hour, global, 1-degree grid meteorological dataset [22,23]. In this study, the hourly initial and boundary conditions were simulated by the WRF to run the three-dimensional trajectory program for fall armyworm, with a spatial resolution of 30 km. The calculation schemes and model parameters used for WRF were listed in Table 2.

### 2.3. Trajectory Analysis

Trajectory analysis is one of the common and effective methods to determine the origin and landing area of the migratory insects [24], which has been successfully used for many migratory noctuid moths such as *Cnaphalocrocis medinalis* (Güenée) [21,25], *Mythimna separata* (Walker) [26], *S. frugiperda* [22,23,27,28]. In this study, possible source regions of the fall armyworm invading Australia were determined by backward trajectory simulation. The date with fall armyworm moths captured by surveillance trapping was set as trajectory simulation dates, and the trajectories were obtained from three origins, Saibai and Erub Islands and Bamaga.

Based on biological and flight characteristics of fall armyworm, trajectories were calculated with the following parameters: (1) Fall armyworm flies downwind at a high altitude [29,30,31], without considering a directional deflection angle [23]; (2) Other similar-sized noctuid moths have a self-powered flight speed of about 2.5–4 m s^−1^ [23,30]. In this trajectory modeling, the self-powered flight vector of 3.0 m s^−1^ was assumed [22]; (3) The noctuid insects typically migrate at night, taking off at dusk and landing at the following dawn [21,25]. Fall armyworm can continuously fly 12 h every night and mostly fly for three consecutive nights, similar to most other noctuid moths whenever flying over land [32]; (4) While the fall armyworm flies over sea, nocturnal continuous flight duration is extended until it reaches land [22]. Therefore, when fall armyworm migrated in the archipelago of northwestern Australia, the backward trajectories were set as the 2 times flight and a single flight lasted for but did not exceed 36 h [22]. Then, the trajectories effective endpoints were selected by the departure time at taking off time and in a host planting region or at least from a terrestrial location [22]; (5) Radar observations show that moths usually fly in a low-level jet stream at altitudes with wind speeds greater than 10 m s^−1^ [29,33]. However, the most appropriative flight height of fall armyworm before trajectory simulating was not determined. In this study eight possible different initial altitudes of 500, 750, 1000, 1200, 1500, 1750, 2000, and 2250 m above mean ground level (AGL) were assumed [22,23,24,25,26,27,28,29,30,31,32,33,34]; and (6) Preliminary flight ability test also found that the fall armyworm stopped flying after a short period when the ambient temperature reached approximately 13.8 °C (Chen H from Nanjing Agricultural University, unpublished data). So fall armyworm cannot fly when the air temperature at flight altitude falls below 13.8 °C, the minimum temperature for survival of fall armyworm [22,23]. The trajectories of the fall armyworm using meteorological conditions at different flight altitudes were simulated.

### 2.4. Synoptic Weather Condition Analysis

Wind-borne migration, in which migrants ascend to altitudes at which they are transported downwind, are governed by synoptic weather patterns [35,36,37]. The direction of the fall armyworm’s migratory flight is highly correlated with wind headings. Their migration can be terminated by atmospheric factors such as downdrafts, rain and cold temperatures, and the moth also probably ends flight through exhaustion [28,38]. Based on meteorological data of NCEP, the distributions of monthly average wind speed and wind direction frequency at 800 hPa, 825 hPa, 850 hPa, 875 hPa, 900 hPa from January to December 2010–2019 from dusk (20:00) to dawn (05:00) at Saibai Island, Erub Islands, and Bamaga were calculated, and the influence of wind speed and wind direction frequency in different seasons on the migration and landing of fall armyworm were analyzed using GrADS 2.1 (version 2.1, https://sourceforge.net/projects/opengrads/files/grads2/2.1.0.oga.1, accessed on 15 February 2021) and R 3.6.2 (version 3.6.2, https://cran.r-project.org/, accessed on 20 May 2021). The average wind speed, temperature, cumulative overnight rainfall at 850 hPa (approximately 1500 m from the ground) during January, 2020 were also extracted to analyse the influences of weather conditions on the flying and landing for fall armyworm.

## 3. Results

### 3.1. Surveillance Trapping and Inferred Migration Dates of Fall Armyworm

According to the surveillance trapping results, three and four fall armyworm moths were first detected in surveillance traps on the islands of Saibai and Erub, in the Torres Strait on 20 January 2020. On 31 January 2020, one fall armyworm female adult was detected at Bamaga, in the northern part of Queensland, Australia. The trapped specimens were confirmed to be fall armyworm by morphological and molecular diagnostics, which became the first identification of fall armyworm in Australia. The different possible immigration periods of fall armyworm in surveillance traps were assumed (Saibai and Erub Islands during 14 to 18 January 2020; Bamaga during 26 to 30 January 2020).

### 3.2. Source Region of Fall Armyworm Identified by Backward Trajectories

To find out the possible source region of fall armyworm immigrants invading Australia, backward trajectories were calculated hourly on probable invading dates of fall armyworm to Saibai and Erub Islands and Bamaga. Saibai and Erub Islands have similar climates, and the collecting dates of captured fall armyworm moths at these sites overlap, thus, their insect sources may be similar. However, the different arrival dates of fall armyworm moth at Bamaga might mean a different source region. During the trajectory analysis, this study identified that the first occurrence of fall armyworm in Australia most likely resulted from migration from Sulawesi Island to the Maluku Islands and then migration to the Torres Strait and Bamaga. Thus, the exact arrival date was estimated using the starting date of valid trajectories, and these dates showed that fall armyworm moths migrated into Australia with two migration waves.

#### 3.2.1. Backward Trajectories Analysis in Saibai and Erub Islands

**Figure 1:**
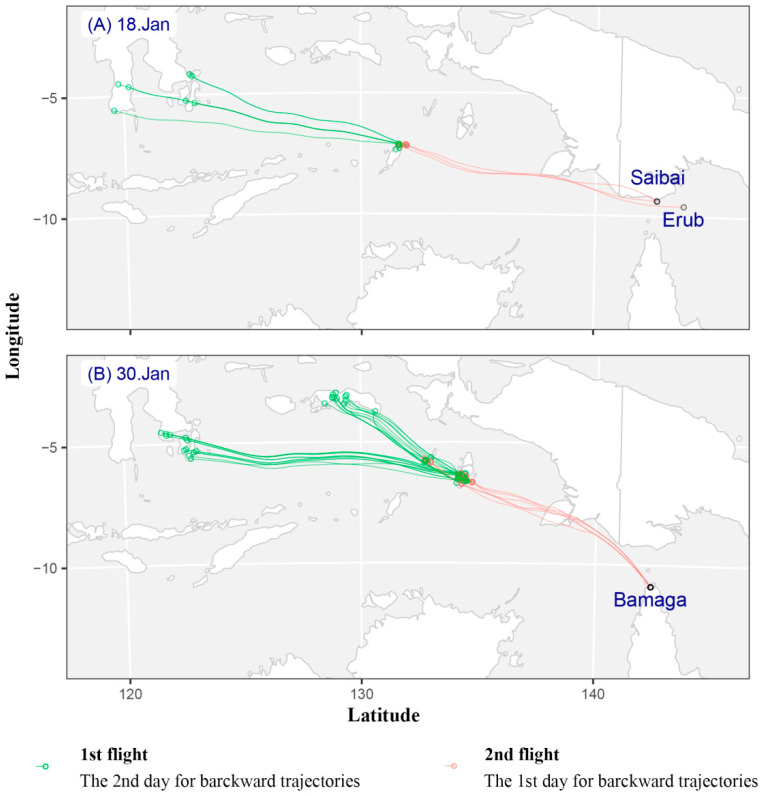
Simulated effective backward trajectories showed the possible source and migrating pathway of *S. frugiperda*, which was found in Australia in 18 and 30 January 2020. Note: Green and red circle represent backward trajectory end-point for the 2nd day and the 1st day, respectively. (**A**) On 18 January 2020, (**B**) On 30 January 2020.

This study analyzed backward trajectories from Saibai and Erub Islands on the probable arrival date of fall armyworm (i.e., 15 to 19 January) (Figure S1). Among the trajectories of 15 to 19 January, only the backward trajectory of 18 January is meaningful and realistic (Figure 1A). Seventeen effective trajectories appeared on 18 January 2020 (Figure 1). There were 14 valid trajectories for the first migration and 3 valid trajectories for the second. The first migration of fall armyworm took off in southern Sulawesi Island at 19:00 on the evening of 14 January and landed in Tanimbar Island between 20:00 on 15 January and 05:00 on 16 January. The average flight duration was 15.79 ± 4.03 h, and most of these trajectories were concentrated at the altitude ranged from 750 m to 2250 m (Figure 1A, Table S1). The second migration took off at approximately 19:00 in the evening on 16 January, and landed in the Torres Strait from 21:00 on 17 January to 05:00 on 18 January. The average flight duration was 30.67 ± 2.40 h, and most of these trajectories were concentrated at the altitude ranged from 500 m to 1500 m (Figure 1A, Table S1). In other word, fall armyworm moths from Sulawesi Island of Indonesia took off between 15 January and 16 January, and arrived at Saibai and Erub Islands on 18 January.

#### 3.2.2. Backward Trajectories Analysis in Bamaga

In regards to Bamaga, hourly backward trajectories were calculated for fall armyworm on 29–30 January (Figure S1). There were 70 valid trajectories on 30 January 2020 (Figure 1B). There were 64 valid trajectories for the first migration and 6 valid trajectories for the second. In the first flight, fall armyworm took off in southeastern Sulawesi Island at approximately 19:00 in the evening of 26 January and landed in Aru Island between 20:00 on 27 January and 01:00 on 28 January. The average flight duration to cover this migration distance was 14.55 ± 1.80 h. The suitable flight altitude range is 500–2250 m (Figure 1B, Table S1). The second migration took off at approximately 19:00 in the evening on 28 January, and landed in Bamaga from 20:00 on 29 January to 01:00 on 30 January. The average flight duration was 28.00 ± 0.73 h. The suitable flight altitude range is 500–750 m (Figure 1B, Table S1). Thus, the effective source region of fall armyworm moths is Aru Island and southeastern Sulawesi Island, Indonesia.

### 3.3. Synoptic Weather during Fall Armyworm Migration Period

**Figure 2:**
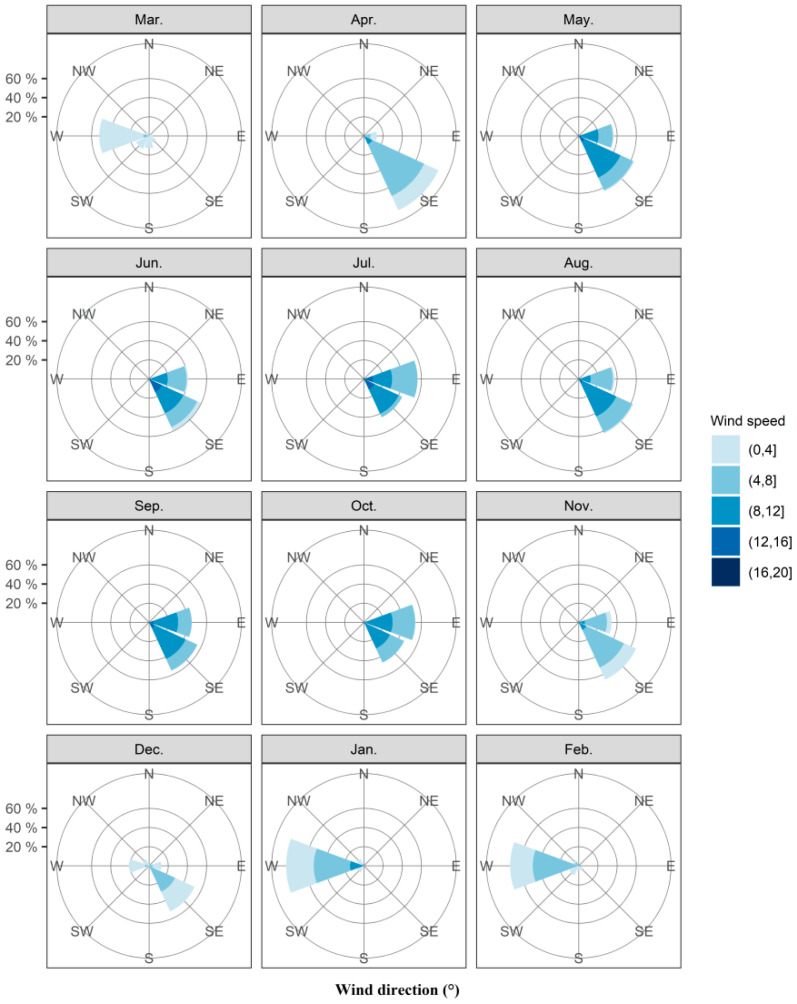
Circular histograms of monthly mean wind directions at 800–900 hPa during 20:00 to 05:00 on Saibai and Erub Islands and Bamaga from 2010 to 2019. Note: The area of the color segments is proportional to the number of occasions when wind directions fell within each 22.5° bin. There are 1500 points in each histogram (3 locations, 5 altitudes, 10 h, 10 years).

In northern Australia, onset of the monsoon winds is conducive to the invasion of fall armyworm into Australia. Thus, we have counted the wind fields over Saibai, Erub, and Bamaga in the past ten years (Table S2). In the decade from 2010 to 2019, strong westerly winds at 800–900 hPa (approximately 1000–2000 m above sea level) occurred only in January and February, the probability being 79.20% and 74.84% respectively. The average wind speed in these 2 months was 5.35 m s^−1^ and 4.37 m s^−1^, with strong monsoon winds over 8 m s^−1^ also record in January. At the same time, the probability of northwest wind in January and February was 15.13% and 11.60% respectively. The average wind speed in these 2 months was 3.58 m s^−1^ and 4.04 m s^−1^ (Figure 2, Table S2). Hence, fall armyworm is highly likely to invade Australia across the Timor Sea with the assistance of dominant westerly winds in January and February, and January is the most likely month. Moreover, while westerly and northwesterly wind also prevailed from March and December (Figure 2, Table S2), the average wind speed was less than 3 m s^−1^, which cannot provide sufficient carrier airflow for the long-distance migration of fall armyworm. Easterly and northeasterly winds prevail from April to November (Figure 2, Table S2), fall armyworm cannot successfully travel to Australia with this wind direction. Thus, the high risk period for invasion of fall armyworm is only likely to have occurred in January–February due to monsoon winds. 

**Figure 3:**
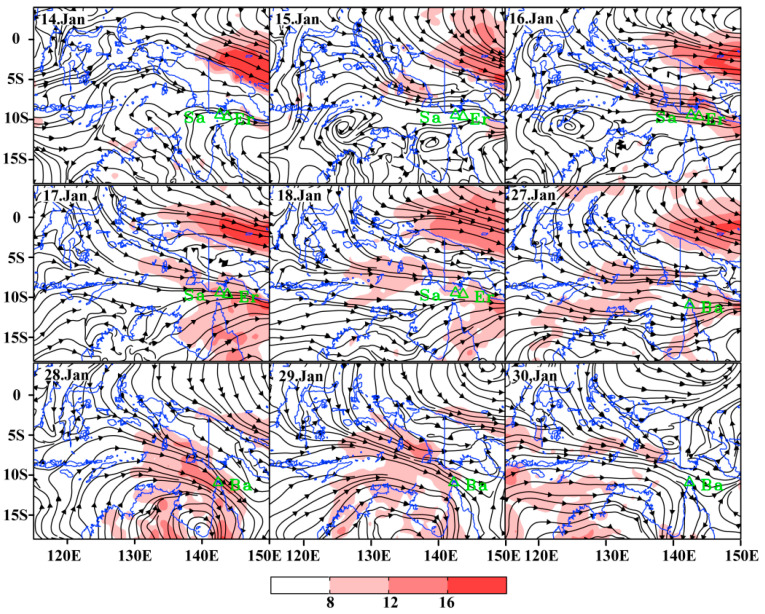
The wind pattern at the level of 850 hPa (approximately 1500 m above sea level) during the migration period. Note: Sa represents Saibai Islands, Er represents Erub Islands, Ba represents Bamaga.

The influences of weather conditions on the migrating and landing for fall armyworm during January 2020 were further analyzed. On 14 January, wind direction in the Torres Strait was scattered and the wind speed was less than 8 m s^−1^. The wind speed at Saibai and Erub Islands was only 3.31 m s^−1^ and 3.96 m s^−1^ respectively. Strong westerly wind from southeast Indonesia to the Torres Strait commenced since 15 January. The wind speed at Saibai and Erub Islands increased to 11.23 m s^−1^ and 10.41 m s^−1^ on 16 January, and wind speed above 10 m/s remained during 17 to 18 January (Figure 3). Similarly, a strong westerly wind prevail in the Torres Strait due to the influence of a tropical cyclone from 27 to 29 January, with average daily wind speeds of 10.08 m s^−1^, 13.38 m s^−1^ and 9.51 m s^−1^, respectively (Figure 3). Therefore, there was suitable carrier airflow in the Torres Strait during 16 to 18 January and 27 January to 29, to enable fall armyworm to complete a long-distance migration across the sea.

**Figure 4:**
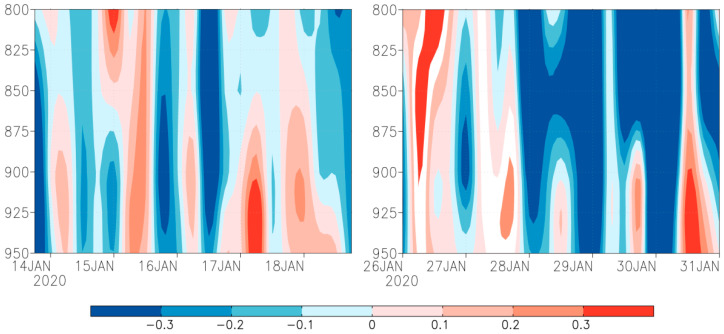
Time-height profile of vertical velocity (Pa/s) from 800 to 950 hPa on Saibai and Erub Islands (**Left**) and Bamaga (**Right**). Note: filled color show wind speed, red represents downdraft, blue represents updraft.

This study also identified that downward flow above 0.3 Pa s^−1^ was present in the periods 17–18 January and 30–31 January (Figure 4). However, the role of downdraft was insignificant, because the downdraft was not strong enough to overwhelm fall armyworm moth’s self-powered flight speed. Hence, downdraft was not the fundamental reason of the landing of the fall armyworm at Torres Strait islands and Bamaga, Australia.

## 4. Discussion

The Torres Strait islands lie in a vast area of the South Pacific sitting between Indonesia, Papua New Guinea and the Australia mainland. These islands, both inhabited and uninhabited, provide ideal stepping-stones for the dispersal of migratory insects [39,40]. Short-distance dispersal events are both more frequent and across a broader season for the short distance between southern Papua New Guinea and the islands of the Torres Strait [41]. However, long-distance migrants moving into Australia may be relatively infrequent and transitory [42,43]. Several studies of insect migration movements in this area are limited, and are skewed towards Culicoides (Diptera) [18,41], Mosquitoes (Diptera) [44], and Butterfly (Lepidoptera) [40]. Recent modelling work has shown that long-distance dispersal was a plausible means of introduction of Culicoides into northern Australia [41,45,46]. So the patterns of dispersal, which are driven by seasonal winds, are likely to be applicable to other migratory insects, such as fall armyworm.

Establishment and spread requires not just a dispersal event, but the “successful” combinations of suitable host, meteorological and environmental factors at both source and invasion places [44]. In this study, the migratory paths and wind systems for the case of fall armyworm first invading Australia were analyzed. The migration trajectory simulation demonstrated fall armyworm immigrants in Torres Strait and Bamaga mainly came from Sulawesi and Moluccas Islands of Indonesia. According to the investigation results of the Directorate of Plant Protection in the Ministry of Agriculture, Indonesia, fall armyworm was first detected in West Sumatra, Indonesia in March 2019 [16], and the pest has rapidly spread across Sumatra, Java, East Nusa Tenggara, Kalimantan, Sulawesi, and Maluku (personal communication). Fall armyworm has infested corn field right across Indonesia due to its long-distance migration. Hence, Sulawesi, and Maluku Islands, Indonesia, highlighted as important regional source sites for Australia, could provide enough fall armyworm immigrants. Fall armyworm could invade in northern parts of Australia from Indonesia in January–February every year due to monsoon winds. This is consistent with previous studies about long-distance aerial dispersal modelling of Culicoides [45,46]. However, as fall armyworm was first found in Timor-Leste and Papua New Guinea after February 2020, these regions could not have been effective source sites for the initial Australian fall armyworm invasion.

Natural insect migration, known as wind-borne migration, is significantly influenced by a variety of atmospheric processes [35]. Due to their limited flight capabilities and small bodies, most migratory insects take advantage of the seasonal atmospheric characteristics undertaking regular long-distance migrations, tracking seasonal changes in resources and habitats [36]. Flying at high altitude, and landing of migratory insects, are influenced by meteorological factors and associated phenomena [47,48]. Several studies have shown the low-level jet stream can provide suitable flight conditions for the long-distance migration of fall armyworm [28,34,38]. Similar conclusions also appear in this study. Based on the actual invasion period of fall armyworm into Australia, wind speed above 10 m/s remained in the Torres Strait during 16 to 18 January and 27 to 29 January, which provides suitable carrier airflow to enable fall armyworm to complete a long-distance migration across the sea. Moreover, the wind systems during 2010–2019 were analyzed: strong westerly winds occurred only in January and February. With the assistance of dominant monsoon winds, the invasion of fall armyworm is likely to have occurred in January–February, which is in keeping with the previously determined ‘high-risk’ period for the invasion of migratory insects [41].

The landing of migratory insects is also influenced by meteorological factors and phenomena, like flying at a high altitude [49]. Some specific weather conditions will force the migratory insect population to land, forming a local large-scale population, among which the downdraft and rain scouring are the main reasons for the massive forced landing of insect population [48]. Similarly, some atmospheric factors such as low temperature barriers, wind shear, rainfall and downward flow can cause a forced landing of fall armyworm [28,38]. In this study, downdraft airflow above 0.3 Pa s^−1^ appeared in the immigrating areas of the Torres Strait in the periods 17–18 January and 30–31 January. However, the weak downdraft was not strong enough to overwhelm the fall armyworm moth’s self-powered flight speed. Therefore, this study infers that downdraft was not the fundamental reason of the landing of fall armyworm at Torres Strait islands and Bamaga, Australia.

The long-distance migration capability appears to be the key driver for the rapid expansion and the large-scale outbreak of fall armyworm globally [10]. With the aid of high-altitude wind, fall armyworm moth can fly long distances covering hundreds of kilometers over several nights [49]. Due to the strong long-distance migration ability of fall armyworm, its surprisingly rapid spread and its significant capacity to generate high yield losses has resulted in it attracting wide concern worldwide [5,7,8]. Fall armyworm had overcome the challenges of geographic barriers, invading in northern parts of Australia in 2020 [17]. Currently, fall armyworm has also been found in the Northern Territory, New South Wales, Western Australia and Victoria, as well as spreading to Tasmania, the southernmost island in Australia. Eradication is considered to be no longer feasible as fall armyworm has established local population and further range expansion in Australia is likely. Fall armyworm could form a regular, seasonal round-trip migration between northern Australia and southern parts of Australia. From spring to autumn, fall armyworm moth will most likely migrate southward and infest areas of production in most parts of Australia, putting greater pressure on crop production. Therefore, understanding the migration paths and the possible source regions of fall armyworm immigrants will be useful for the early warning and management of fall armyworm within Australia.

## 5. Conclusions

Fall armyworm is recognized as one of most highly destructive agricultural pests, which has recently invaded Australia in 2020. Despite some uncertainties, this study provides much-needed insights into source regions of the first immigration of fall armyworm invading Australia. This research on the simulated migration paths elucidated the effective source regions of newly-invaded fall armyworm in the islands of Saibai and Erub and Bamaga. This study has confirmed that the immigration of fall armyworm to Australia was most likely from Sulawesi and Moluccas Islands of Indonesia. The high invasion risk period of fall armyworm was also confirmed to be January–February. These predictions can allow the Australian government and biosecurity departments to tailor their strategies for the future monitoring and managing of fall armyworm. 

## Figures and Tables

**Table 1:** Surveillance trap placement of *S. frugiperda* in areas of high invasion risk of Australia.

Trap Name	Location	Latitude	Longitude	Trap Type
FAW-Erub001	Sewerage treatment plant, Erub Island	−9.590167	143.75698	Unitrap
FAW-Erub002	Northeast of water storage, Erub Island	−9.5915	143.77208	Unitrap
FAW-Saibai001	East of cemetary, Saibai Island	−9.382222	142.6075	Unitrap
FAW-Saibai002	Southern end of airstrip, Saibai Island	−9.381111	142.62528	Unitrap
FAW-Bamaga001	Sagaukaz Street, Bamaga	−10.89583	142.38442	Unitrap
FAW-Bamaga002	Koraba Road, Seisia	−10.84815	142.36731	Unitrap
FAW-Badu001	Blanket Yabu Street, Badu Island	−10.15776	142.1682	Unitrap
FAW-Badu002	Esplanade, Badu Island	−10.16836	142.16694	Unitrap

**Table 2:** Selection of scheme and parameters of the WRF model.

Item	Domain 1
Location	10° S, 132° E
The number of grid points	130 × 150
Distance between grid points	30
Layers	30
Map projection	Mercator
Microphysics scheme	WSM6
Longwave radiation scheme	RRTMG
Shortwave radiation scheme	RRTMG
Surface layer scheme	Monin-Obukhov
Land/water surface scheme	Noah
Planetary boundary layer scheme	YSU
Cumulus parameterization	Tiedtke
Forecast time	72 h

## Data Availability

All data is available in this paper.

## Supplementary Materials

The following are available online at https://www.mdpi.com/article/10.3390/insects12121104/s1. Figure S1: Simulated backward trajectories showed the possible source and migrating pathway of *S. frugiperda* which found in Australia in January 2020, Table S1: Valid trajectories of *S. frugiperda* for the Australia migration case, Table S2: Statistics for wind direction and wind speed at 800–900 hPa during 20:00 to 05:00 in Saibai and Erub Islands and Bamaga from 2010 to 2019.
