# Supplementary material for: Source Regions of the First Immigration of Fall Armyworm, Spodoptera frugiperda (Lepidoptera: Noctuidae) Invading Australia
Source: Insects. 2021 Dec 10;12(12):1104. doi: 10.3390/insects12121104 (PMC8704567; doi:10.3390/insects12121104)
Supplement: Supplementary file 1 [file insects-12-01104-s001.zip › insects-1467427-supplementary.pdf]

Supporting Information

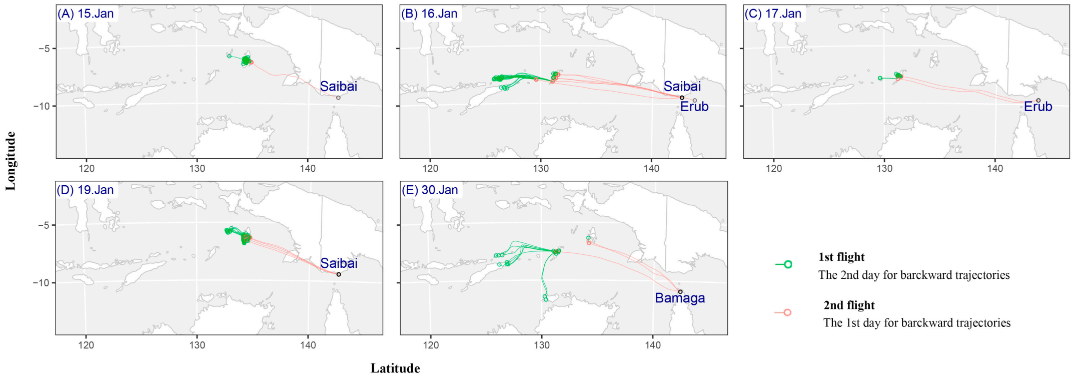

Figure S1. Simulated backward trajectories showed the possible source and migrating pathway of *S. frugiperda* which found in Australia in January, 2020. Note: Green and red circle represent backward trajectory end-point for the 2nd day and the 1st day.

Table S1. Valid trajectories of *S. frugiperda* for the Australia migration case.

Location	Nights	Number of valid trajectories	Probable arrival date	Simulated flight duration of trajectories (hours ± S.E.)	Height of trajectories (above sea level, m)	Arrive date and time - start date and time
Saibai&Erub	1	3	18 Jan.	30.67±2.40	500-1500	17.Jan 21:00~18.Jan 05:00 - 16.Jan 19:00
Saibai&Erub	2	14	18 Jan.	15.79±4.03	750-2250	15.Jan 20:00~16.Jan 05:00 - 14.Jan 19:00
Bamaga	1	6	30 Jan.	28.00±0.73	500-750	29.Jan 20:00~30.Jan 01:00 - 28.Jan 19:00
Bamaga	2	62	30 Jan.	14.55±1.80	500-2250	27.Jan 20:00~28.Jan 01:00 - 26.Jan 19:00

Table S2. Statistics for wind direction and wind speed at 800- 900 hPa during 20:00 to 05:00 in Saibai and Erub Islands and Bamaga from 2010 to 2019.

Month	Wind direction frequency and average wind speed							
	West		Northwest		Southeast		East	
	Proportion	mean wind speed	Proportion	mean wind speed	Proportion	mean wind speed	Proportion	mean wind speed
January	79.20%	5.35	15.13%	3.58	5.00%	0.38	0.27%	4.37
February	74.87%	4.37	11.60%	4.04	5.00%	1.7	1.73%	1.11
March	53.20%	2.49	11.67%	1.82	5.33%	1.43	6.50%	2.49
April	/	/	/	/	66.07%	5.58	33.60%	4.92
May	/	/	/	/	47.27%	9.48	52.73%	8.72
June	/	/	/	/	46.20%	9.61	53.80%	8.08
July	/	/	/	/	34.53%	10.79	65.27%	8.93
August	/	/	/	/	50.27%	8.85	49.73%	7.52
September	/	/	/	/	42.27%	9.27	57.73%	9.17
October	/	/	/	/	24.33%	8.07	75.67%	8.2
November	/	/	/	/	43.80%	5.29	56.20%	5.74
December	17.60%	3.01	14%	2.34	41.40%	4.03	19.80%	3.45

Note: '/' represents no data in this sub-category.
